# Blood Transcript Profiling for the Detection of Neuroendocrine Tumors: Results of a Large Independent Validation Study

**DOI:** 10.3389/fendo.2018.00740

**Published:** 2018-12-04

**Authors:** Mark J. C. van Treijen, Catharina M. Korse, Rachel S. van Leeuwaarde, Lisette J. Saveur, Menno R. Vriens, Wieke H. M. Verbeek, Margot E. T. Tesselaar, Gerlof D. Valk

**Affiliations:** ^1^Department of Endocrine Oncology, University Medical Center Utrecht, Utrecht, Netherlands; ^2^Center for Neuroendocrine Tumors, ENETs Center of Excellence, Netherlands Cancer Institute, University Medical Center Utrecht, Utrecht, Netherlands; ^3^Department of Clinical Chemistry, The Netherlands Cancer Institute, Amsterdam, Netherlands; ^4^Department of Gastroenterology, The Netherlands Cancer Institute, Amsterdam, Netherlands; ^5^Department of Endocrine Surgical Oncology, University Medical Center Utrecht, Utrecht, Netherlands; ^6^Department of Medical Oncology, The Netherlands Cancer Institute, Amsterdam, Netherlands

**Keywords:** biomarker, multigene transcripts, neuroendocrine tumors, gastroenteropancreatic, NET, chromogranin A, carcinoid

## Abstract

**Background:** Available neuroendocrine biomarkers are considered to have insufficient accuracy to discriminate patients with gastro-entero-pancreatic neuroendocrine tumors (GEP-NETs) from healthy controls. Recent studies have demonstrated a potential role for circulating neuroendocrine specific transcripts analysis—the NETest—as a more accurate biomarker for NETs compared to available biomarkers. This study was initiated to independently validate the discriminative value of the NETest as well as the association between tumor characteristics and NETest score.

**Methods:** Whole blood samples from 140 consecutive GEP-NET patients and 113 healthy volunteers were collected. Laboratory investigators were blinded to the origin of the samples. NETest results and chromogranin A (CgA) levels were compared with clinical information including radiological imaging to evaluate the association with tumor characteristics.

**Results:** The median NETest score in NET patients was 33 vs. 13% in controls (*p* < 0.0001). The NETest did not correlate with age, gender, tumor location, grade, load, or stage. Using the cut-off of 14% NETest sensitivity and specificity were 93 and 56%, respectively, with an AUC of 0.87. The optimal cut-off for the NETest in our population was 20%, with sensitivity 89% and specificity 72%. The upper limit of normal for CgA was established as 100 μg/l. Sensitivity and specificity of CgA were 56 and 83% with an AUC of 0.76. CgA correlated with age (rs = 0.388, *p* < 0.001) and tumor load (rs = 0.458, *p* < 0.001).

**Conclusions:** The low specificity of the NETest precludes its use as a screening test for GEP-NETs. The superior sensitivity of the NETest over CgA (93 vs. 56%; *p* < 0.001), irrespective of the stage of the disease, emphasize its potential as a marker of disease presence in follow up as well as an indicator for residual disease after surgery.

## Introduction

Neuroendocrine tumors (NETs) of the gastro-entero-pancreatic tract (GEP-NETs) are a heterogeneous group of neoplasms with unpredictable and diverse biological behavior patterns (1). The prevalence of NETs has increased over the past 30 years, due to an increased incidence and improved survival (2–4). There is no consensus in the NET community on the use of available biomarkers for early diagnosis and disease monitoring during treatment (5–7). As such, the focus in NET research has moved toward the molecular pathology, molecular imaging, and biological behavior of NETs (8–10). In this evolving landscape, identification of sensitive and specific biomarkers that provide real-time pathobiological information, is considered the most clinically promising approach (6).

The National Institutes of Health (NIH) defines strict criteria for the efficacy of a biomarker (11). These are not met by existing neuroendocrine biomarkers as they have insufficient accuracy to indicate the presence of disease, identify tumor aggressiveness, or determine responsiveness to treatment. The inherent biological variability of NETs inevitably leads to differences in secretion of peptides and amines, thus limiting the clinical utility for most biomarkers in a NET population (1, 12, 13). Some examples of current NET biomarkers are insulin, gastrin, serotonin, pancreatic polypeptide (PP), and Chromogranin A (CgA). CgA is the most accepted biomarker for NET in current practice (12), although it is not without significant shortcomings. The most important of these are that CgA is expressed in healthy tissue and can be falsely elevated due to systemic inflammation or secondary to proton-pump inhibitors (PPIs) use (14, 15). Beyond this, the direct correlation between tumor volume and CgA levels as well as variability of existing CgA immunoassays further limits CgA as a reliable biormarker for NET (13, 16, 17).

These shortcomings emphasize the need for a new and more reliable biological tool to provide enhanced information regarding presence of disease and disease status in NETs. The clinical utility of circulating transcripts as biomarkers for a multitude of solid tumors in general oncology has previously been demonstrated (18–22). Recent studies have shown promising results on circulating transcripts analysis as a new biomarker panel for NETs (23–26). This blood-derived multianalyte assay, NETest (Wren Laboratories, Branford, CT, USA) measures gene expression of 51 circulating NET marker genes simultaneously by q-PCR (25) and turned out to outperform CgA measurement as a diagnostic marker (24).

Before the NETest can be introduced in clinical practice, the independent assessment of the test characteristics and possible pitfalls is required. Formal validation of the NETest in terms of its discriminative value in a truly independent and representative patient cohort is essential because most biomarkers are confined by the heterogeneity of NETs. This study was therefore designed to assess the value of the NETest for discriminating between consecutive GEP-NET patients and healthy volunteers. Sensitivity, specificity, and area under the curve of the NETest were determined with reference to healthy individuals in the Netherlands. As a comparator biomarker, CgA was used. Additionally, we evaluated whether NETest could predict the origin of the tumor, the tumor load, or tumor grade. In combination, these independent assessment of the NETest should guide future research and implementation of NETest as a potential biomarker.

## Subjects and Methods

### Subjects

Patients with histologically proven, well-differentiated sporadic NETs and healthy volunteers as controls were approached for inclusion between March 2014 and March 2017 at the Netherlands Cancer Institute (NCI) (Amsterdam, The Netherlands). Patients were excluded if no imaging was available within 6 months before or after the NETest, if they had another malignancy or exhibited no detectable disease on imaging studies. Central pathology review was performed at the NCI.

Volunteers, non-related subjects accompanying patients at the outpatient clinic, were included if there was no known malignancy present at the time of blood draw and they did not exhibit any physical complaints.

NETest results and CgA levels were compared with concordant radiological imaging, i.e., computed tomography (CT), ultrasound (US), or magnetic resonance imaging (MRI) to evaluate if either of the biomarkers corresponded with tumor load or tumor location. Outcomes of functional imaging (^68^Ga-DOTATATE PET/CT, or somatostatin receptor scintigraphy with ^111^In-pentetreotide (SRS) were used in cases in which radiological imaging was not available. Concordant imaging was considered as the reference standard for tumor load. The absolute number of metastasis, the size of largest objectivized tumor mass and the total number of affected organs by metastatic disease were extracted from the radiology reports and used as markers for tumor load.

NETs were graded according to the World Health Organization (WHO) 2017 grading system (27). Ethics committee approval (NCI, Amsterdam) was obtained.

### Test Methods

#### Samples Collection

Blood samples (6 ml; peripheral blood) were collected in EDTA tubes after written informed consent was obtained. After collection, samples were thoroughly mixed and immediately stored on ice. They were coded and stored at −80°C within 2 h after collection according to standard molecular diagnostics protocols for PCR-based studies (28). Serum for CgA analysis was collected at the same time. CgA levels were determined at the NCI (Clinical Laboratory). Blood samples for both healthy volunteers and NET patients were sent for NETest at Wren Laboratories, Connecticut, USA. All samples were anonymized and coded, and laboratory investigators at both sites were blinded to the origin of the sample, clinical diagnosis, and disease status.

#### PCR-Based Transcript Analysis: NETest

Details of the PCR methodology, mathematical analysis, and validation have previously been published comprising a 2-step protocol (RNA isolation/cDNA production and q-PCR) from EDTA-collected whole blood (23, 25, 29, 30). Target transcript levels are subsequently normalized and quantified vs. a historical (2014) population control (25). Final results are expressed as an activity index or NETest score from 0 to 100% (23). The upper limit of normal (ULN) has previously been set at 14% (31).

qPCR cycle thresholds for the reference gene ALG9 were checked in all our samples (patients and controls). No deviating values were found ruling out possible degradation of transcripts.

#### CgA Measurement

B·R·A·H·M·S Chromogranin A is an automated immunofluorescent assay for the quantitative determination of CgA in human serum using the KRYPTOR instrument (BRAHMS GmbH, Hennigsdorf, Germany). The ULN is established as 100 μg/l (32).

### Statistical Analysis

Statistical analyses were performed using Statistical package for Social Sciences (SPSS) 21. Confidence intervals for metrics were calculated with R. Statistical significance was defined at a *p*-value < 0.05.

To describe clinical characteristics, NETest score and CgA levels, the mean ± standard deviation or median with range were calculated in normal distributed and not-normal distributed data, respectively, (Kolmogorov-Smirnov; K-S). The Mann-Whitney *U*-test and the Wilcoxon signed rank test were used to analyze not-normal distributed continuous variables. Normally distributed continuous data was analyzed using the *T*-test or paired *T*-test. Dichotomous variables were compared with the McNemar or χ2 test.

The discriminating value was expressed by sensitivity and specificity using the area under the Receiver Operating Characteristic curves (AUC) by comparing either the NETest or CgA results of controls and patients with histologically proven GEP-NETs. Relevant positive predictive values (PPV) and negative predictive values (NPV) are given. The McNemar test was used for paired binary data. Receiver operating characteristic (ROC) analysis for NET detection (diagnosis) was constructed. ROC analysis was also used for calculation of cut-offs for the most optimal ULN for the NETest in our population.

Possible differences between categories, like tumor locations and the NETest or CgA were analyzed with Kruskal-Wallis because of the not-normal distribution of both tests (*p* < 0.001). The association with age, gender, tumor location, tumor stage, tumor size, and tumor grade was assessed. Spearman correlation was used for both the NETest and Cga. Tumor stage was defined by two categories: loco-regional disease and distant metastasis. Loco-regional disease was defined as the primary tumor and/or metastasis only in local and/or regional lymph nodes.

## Results

### Study Population

A total of 140 patients with GEP-NETs were included in this study (Table 1). The control population comprised of 113 healthy volunteers. The primary location of the malignancies and the demographics of each group are included in Table 1. One patient had a high proliferative rate (KI-67: 25%) but exhibited well-differentiated morphology and was therefore included and graded as NET grade 3.

**Table 1:** Baseline characteristics of the neuroendocrine tumor patients and control group.

**Characteristic**	**NET**	**Healthy volunteers**	***P*-value**
**Patients**	*N* = 140	*N* = 113	
**Mean age at inclusion**	63 ± 15	52 ± 11	<0.001
**Gender (M:F)**	75:65 (54%:46%)	46:67 (41%:59%)	0.04
**Origin of primary tumor**		N/A	
Appendix	1 (1%)		
Caecum	2 (1%)		
Duodenum	1 (1%)		
Gastric / esophagus	3 (2%)		
Ileum	89 (64%)		
Pancreas	28 (20%)		
Colorectal	5 (3%)		
GEP-NET with unknown origin	11 (8%)		
**Grade**		N/A	
1	91 (65%)		
2	47 (34%)		
3	1 (1%)		
Unknown	1 (1%)		
**Tumor stage**		N/A	
Loco regional disease	8 (6%)		
Distant metastasis	132 (94%)		
**Imaging modality for tumor stage**		N/A	
CT-scan	94 (67%)		
MRI	4 (3%)		
SSRS	12 (9%)		
18F-FDG PET with low dose CT	3 (2%)		
68Ga DOTATATE with low dose CT	26 (19%)		
Ultrasound	1 (1%)		
**Treatment**		N/A	
None	62 (44%)		
Chemotherapy	1 (1%)		
Anti-PD1	1 (1%)		
SSA	73 (52%)		
Everolimus	3 (2%)		
**NETest at baseline**			
Median (range)	33.3 (13–93%)	13,3 (0–80%)	<0.001
**CgA at baseline**			
Median (range)	129 (12–143500)	46 (20–713)	<0.001

*M:F, male/female; NET, neuroendocrine tumor; SSRS, somatostatin receptor scintigraphy (^111^In-DTPA-octreotide); PET, positron emission tomograpghy; 18F-FDG, fluor-18-deoxyglucose; 68Ga DOTATATE, gallium-68 dodecanetetraacetic acid tyrosine-3-octreotate; anti-PD-1, Programmed cell death protein 1 inhibitor; SSA, somatostatin analog*.

### Discriminating Value of the NETest

**Figure 1:**
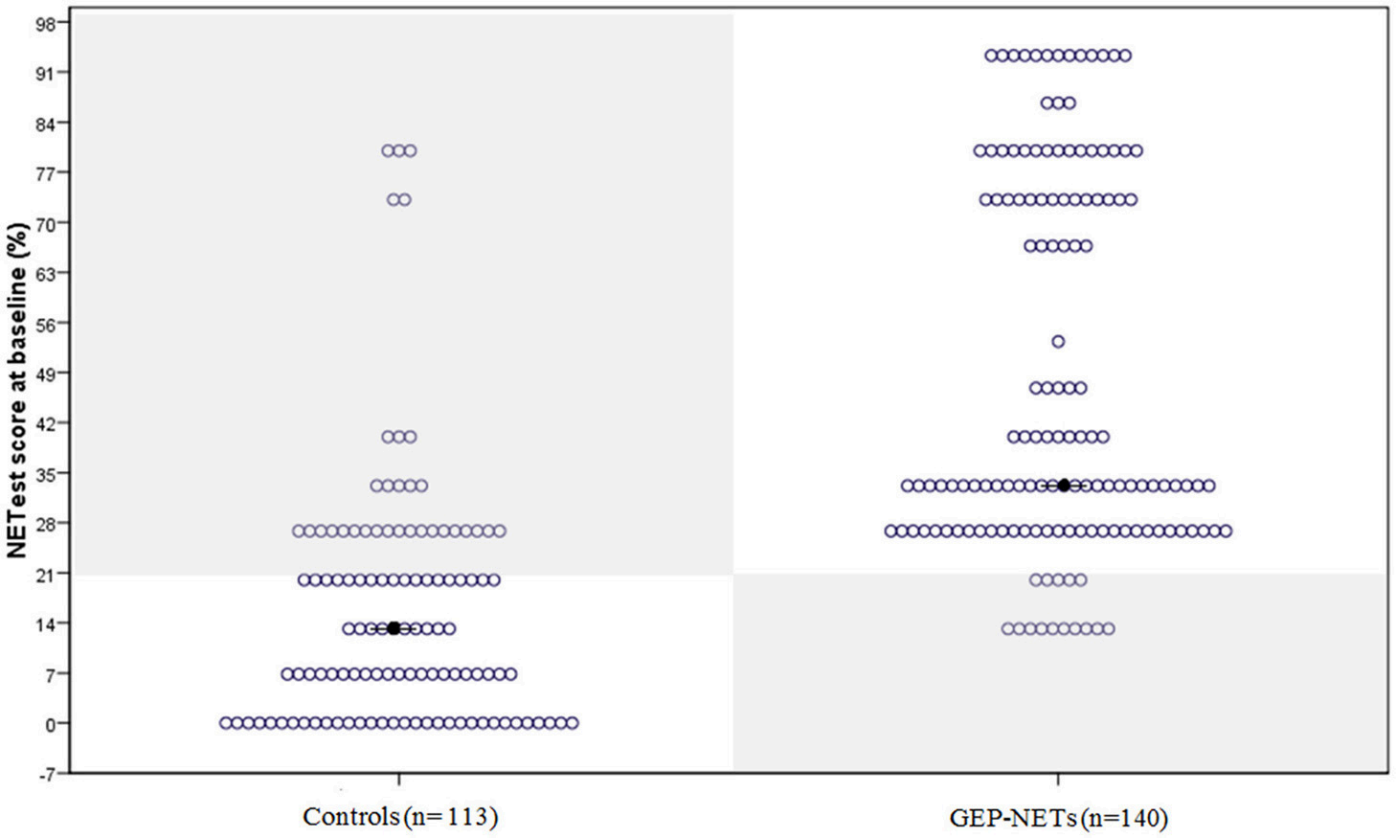
The distribution of the NETest in healthy volunteers and patients with GEP-NETs. The distribution of the NETest results is illustrated in both controls **(Left)** and GEP-NET patients **(Right)**. The results inside the gray squares illustrate deviant results when using the optimal cut-off for our population (20%). The black spots reflect the median NETest outcome. Median NETest outcome in NET patients was 33% compared to 13% in controls (*p* < 0.001).

The median NETest score in patients was 33% (13.3–93%) compared to 13% (0–80%) in controls (*p* < 0.0001). The distribution in patients and controls is illustrated in Figure 1.

**Figure 2:**
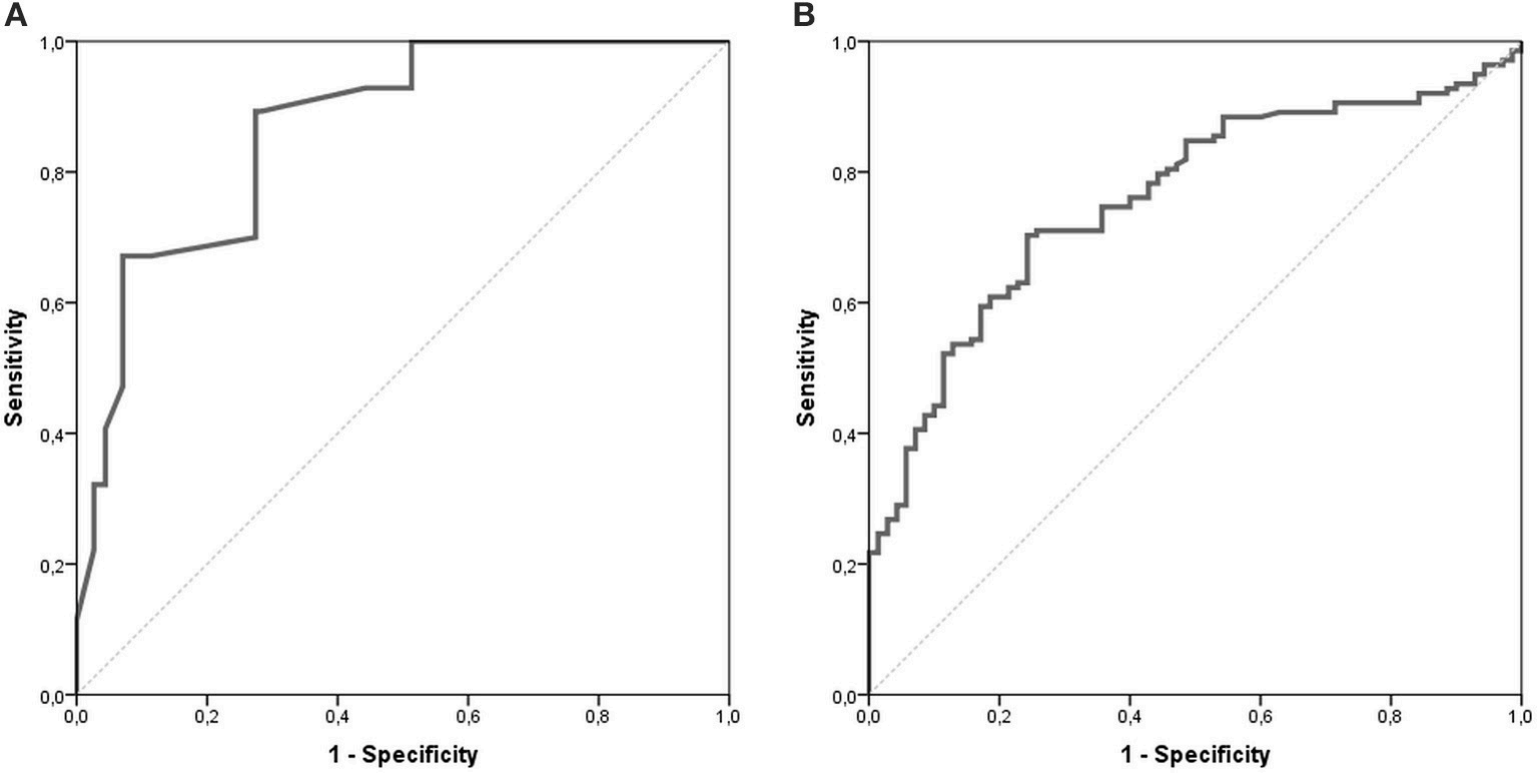
AUROC of the NETest and CgA. The AUC for the NETest is 0.866 [CI 95% 0.822–0.911; **(A)**]. The optimal cut-off for our population was established at 20%. The AUC for CgA 0.759 [CI 95% 0.693–0.825; **(B)]**.

The AUC for the NETest as a diagnostic was 0.87 [95% CI: 0.82–0.91, Figure 2A]. The sensitivity of the NETest was 93% [95% CI: 0.87–0.97] as a diagnostic using the cut-off of 14%. Ten patients with a GEP-NETs (7%) had a false negative NETest score (Table 2). Nine out of these ten negative tests exhibited a grade 1 NET, one was staged as grade 2 (*p* = 0.09). One NETest-negative patient had only loco-regional disease, nine had metastatic disease (*p* = 0.55). Five NETest-negative patients were treated at the time of sampling with SSA (*p* = 0.98). No significant difference in gender, age, or primary tumor location were found between the group patients with false negative NETest scores and patients with true positive test results (*p* = 0.67, *p* = 0.50 and *p* = 0.86) There was no significant correlation between NETest results and age in patients or controls (r_s_ = 0.023, *p* = 0.79 and r_s_ = 0.143, *p* = 0.13, respectively).

**Table 2:** NETest outcome in each group using the cut off of 14%.

	**NET**	**Controls**	**Total**
NETest positive	130	50	180
NETest negative	10	63	73
Total	140	113	253

*NET, neuroendocrine tumor*.

The specificity of the NETest was 56% [95% CI: 0.46–0.65, Table 3]. The NETest was falsely positive in 50 (44%) controls using the cut-off of 14% (Table 2). Both gender and age did not differ between the NETest-positive and -negative controls (Mean age 53 years and 51 years, *p* = 0.402, respectively).The PPV and NPV for the ULN of 14% were 72 and 86%, respectively.

**Table 3:** Metrics of the NETest and CgA.

**Test**	**Sensitivity (%)**	**Specificity (%)**	**PPV (%)**	**NPV (%)**
NETest (ULN 14%)	93	56	72	86
NETest (ULN 20%)	89	72	79	84
NETest (ULN 27%)	67	89	87	67
CgA	56	83	87	49

*The NETest was compared with CgA for detecting GEP-NETs in 140 patients and 113, respectively, 70 controls. Multigene analysis identified GEP-NETs in 93% (N = 130) compared with 56% (N = 77) with CgA (McNemar: p < 0.001). Specificity was significantly better for CgA (83%) compared to the NETest 56% (McNemar: p < 0.001)*.

*ULN, Upper limit of normal*.

Stratification for age and gender showed no significant differences in discriminating value of the NETest. In addition, in subgroup analysis of patients with a median age equal to our control population the outcomes were also the same (sensitivity, specificity, and AUC: 92, 56, and 0.86, respectively).

Sensitivity and specificity were maximal for the cut-off at 20% in our population: sensitivity was 89% (95% CI: 0.82–0.93) and specificity 72% (95% CI: 0.62–0.80) for this threshold (Table 3). Sensitivity and specificity were 67 and 89% for a NETest ULN of 27%. For an ULN of 33%, metrics were 47 and 93%, respectively.

### Discriminating Value of CgA

CgA was measured in 138 patients (2 missing). The median CgA was 129 μg/l [12–143500 μg/l]. CgA levels exceeded the ULN in 77 patients leading to sensitivity of 56% [95% CI:0.47–0.64, Table 3]. Forty-one of sixty-one (67%) patients with negative CgA had a grade 1 GEP-NET (*p* = 0.47). Fifty-six percent of all negative CgA received treatment at time of sampling (*p* = 0.24). There was no statistical difference between false negative and positive CgA for tumor stage, location of tumor or gender, however, age was significantly higher in the positive CgA subgroup (*p* < 0.001).

CgA was measured in *n* = 70 (62%) of the control population. There was no difference in age or gender between controls with CgA results and those without. The median CgA in controls was 46 μg/l [20–713 μg/l]. The specificity of CgA was 83% [95% CI: 0.72–0.91, Table 3]. There was no significant difference in gender and age between the negative and false positive CgA subgroups in the control population. The spearman correlation between CgA and age was significant in both patients and controls with r_s_ = 0.351, *p* < 0.001 and r_s_ = 0.326, *p* = 0.006, respectively. The AUC for CgA was 0.76 [95% CI: 0.69–0.83, Figure 2B].

### NETest vs. CgA

**Figure 3:**
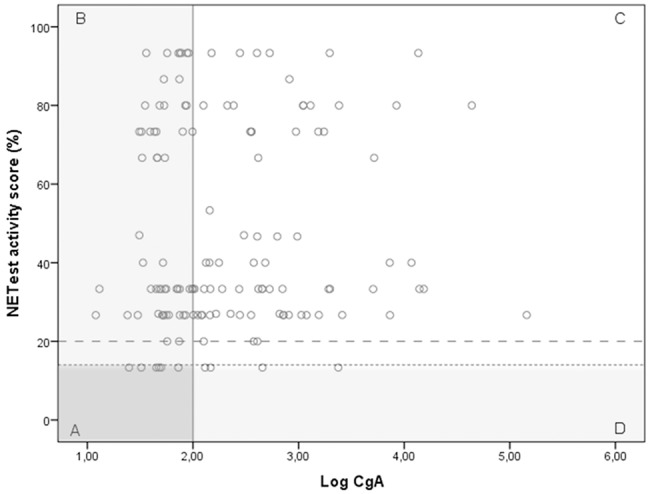
The relationship between the NETest and CgA results for each GEP-NET patient. NETest and CgA results for each patient with proven GEP-NET (*n* = 138). In square A, both test are false negative (*N* = 6). Square B illustrates patients with a false negative CgA but a positive NETest (*n* = 55). In square C, both tests are true positive (*N* = 73). Square D illustrates the false negative NETest (*n* = 4) compared with positive CgA levels. Thus, the multigene analysis identified GEP-NETs in 93% (*N* = 125) compared with only 56% (*N* = 77) with CgA (McNemar: *p* < 0.001) when the suggested cut-off of 14% is respected. There was no correlation between CgA and NETest in the GEP-NET patient group (spearman correlation 0.087; *p* = 0.308) The alternative cut-off is illustrated at NETest activity score 20%. 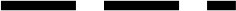
 Cut-off 20%. This is the most optimal cut-off in our population. 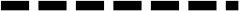 Cut-off 14%. This cut-off is suggested in previous studies.

A significant discordance was identified between NETest and CgA in 59 GEP-NET patients (Figure 3); a better sensitivity was observed for the NETest vs. CgA for identifying GEP-NETs (93% vs. 56%; *p* < 0.001). On the other hand, specificity was significant better in CgA compared to NETest (*p* < 0.001).

CgA was positive in 43% of the patients staged as loco-regional compared with 88% of NETest. This difference was not statistically significant, likely due to small sample size (*N* = 7; McNemar *p* = 0.25). In patients with distant metastasis, the NETest was positive in 93% compared with 56% for CgA (*p* < 0.001).

There was a weak, but significant correlation between CgA and the NETest for our total population (r_s_ = 0.280, *p* < 0.001).

### Association Between NETest and Disease Status

There was no relationship between the NETest activity score and the location of the original tumor (H = 7.60; *p* = 0.474). There was no difference in NETest results between the two largest tumor groups, pancreatic NET, and small bowel NET (*p* = 0.667). No correlation was identified between tumor grade and NETest outcome (r_s_ = −0.036, *p* = 0.676) or tumor stage (r_s_ = 0.103, *p* = 0.225). However, the correlation between the NETest score and tumor stage in imaging subgroups [anatomical imaging (*n* = 99) and functional imaging (*n* = 41)] was weak, but significant in the functional imaging group (r_s_ = 0.360, *p* = 0.02). In this group, NETest scores were significantly higher in those with distant metastases compared with those with loco-regional disease (median 33.3% [13.3–86.7%] vs. median 26.7% [13.3–26.7]; *p* = 0.019). We found no correlation with the absolute number of metastases (r_s_ = 0.077, *p* = 0.39) or tumor size (largest objectivized tumor mass (in mm) reported in a patient; *n* = 96; r_s_ = 0.127, *p* = 0.22) and the NETest. We categorized the total number of metastases in patients into 6 categories (no metastases, solitary metastasis, 2–4 metastases, 5–9 metastases; 10–19 metastases and 20 or more metastases). There was no significant difference (H = 6.35; *p* = 0.27) between these categories and the NETest score. Subgroup analysis in patients with metastatic disease identified no differences between the location of metastases (e.g., presence of liver metastases) and the NETest activity score or the number of affected organs by metastatic disease (metastases in 1–6 organs; H = 2.66; *p* = 0.75). Analysis of the imaging subgroups yielded no significant results for all these surrogate markers.

### Relationship Between CgA and Disease Status

CgA levels exhibited no correlation with the location of the tumor (H = 7.9; *p* = 0.444), the tumor grade (r_s_ = 0.164; *p* = 0.06) or tumor stage (r_s_ = 0.115, *p* = 0.180). The median CgA for patients with grade 1 tumors compared with those with grade 2 tumors was 122 vs. 211, respectively (*p* = 0.07). No significant correlations were identified in imaging subgroups.

A significant correlation between CgA and the absolute number of metastases in a patient (r_s_ = 0.428, *p* < 0.001), tumor size (r_s_ = 0.356; *p* < 0.001) and the six categories of metastases as described above (r_s_ = 0.447, *p* < 0.001), was found. CgA was also higher in patients with metastatic disease in multiple organs (H = 17.3; *p* = 0.004).

## Discussion

This is the largest cohort to date in which the NETest was independently evaluated, including a variety of GEP-NET patients representative of daily clinical practice. All samples were collected according to the same protocol and samples from patients and controls were blinded for all researchers to prevent observer bias. Furthermore, a direct comparison between the NETest and CgA was performed. Both comparators were measured at the same time in the same patient with histopathology as gold standard for diagnosis.

NETest sensitivity was significantly higher to CgA, the current standard biomarker. In contrast, the specificity of CgA was higher. Furthermore, we noted that the sensitivity of CgA in our population was significantly lower to that previously reported in the literature; published sensitivities ranging from 32 to 93% with an overall sensitivity of 73% in a meta-analysis (15). This illustrates the wide variation of CgA between study populations, assays, and laboratories. We did not identify an association with tumor grade or tumor stage for either the NETest or CgA. However, a significant correlation between NETest scores and tumor stage was found in the analysis of tumors evaluated by functional imaging. In line with previous literature, CgA correlated with all surrogate markers for tumor load whereas the NETest showed no correlation. There was no difference between the locations of the primary tumors and NETest levels.

The superior sensitivity of the NETest compared to CgA, irrespective of the tumor stage or tumor grade, might be explained by (1) the dependency of CgA on neuroendocrine cell type and its secretory activity, and (2) the breadth of tumor biology covered by the multi-transcriptome assay approach (NETest). Different neoplastic processes can lead to altered gene expression patterns with heterogeneity in tumors as a result. A multiple synchronous transcript analysis may provide better diagnostic and predictive information than a single secretory protein such as CgA. The superior diagnostic sensitivity confirms the wider detecting range for this heterogeneous group of tumors. It may even have a role as a potential biomarker in treatment follow up or surveillance in GEP-NET patients, but this was not addressed in this study.

The specificity of the NETest in this study is lower when compared to previous reports (24, 30). This might be explained by several factors. The NETest has evolved from a discriminating biomarker initially established in 2013 (25, 30), to a biomarker panel aiming to provide information about the course of disease and treatment efficacy, developed in 2015 (23, 26, 33). We should also consider that upregulation of gene expression could be a reflection of non-malignant processes and would therefore affect the specificity of this test. The selection of the 51 marker genes is based on significant differences in expression between GEP-NET patients and controls from multiple GEP-NET gene panels (tissue based-, blood based-, and literature curated panel of genes). Although the blood transcripts correlated with tumor tissue expression levels (25), some molecular pathways involved in cancer development can also be upregulated in stress or inflammation (34). For example, KRAS, which is one of the 51 marker genes, can be activated due to DNA damage by free radicals during inflammation leading to a higher expression levels (35). This is also reflected by studies detecting mutant KRAS in free circulating DNA in cancer-free subjects, indicating that the activation of KRAS is not a specific indicator of malignancy and not suitable as an isolated biomarker for tumor detection (36). This may result in a false positive test, i.e., the detection of non-GEP-NET samples as a “NET,” reducing the specificity of the test. Consequently, the outcome of the NETest in other malignancies, especially those with possible neuroendocrine differentiations [e.g., colorectal carcinoma or prostate tumors (37, 38)] need to be considered in future studies.

Degradation of extracellular circulating mRNA could theoretically lead to both false negative results (degradation of target genes mRNA) or false positive results (degradation housekeeping genes mRNA) and therefore alter both sensitivity or specificity (39, 40). In a previous study, extracellular RNA was stable up to 3 h (41). We found no deviating values of housekeeping genes in our samples. This suggests that the sensitivity and specificity are accurate in the current study.

The specificity of CgA was similar to that previously reported (15). The high specificity is inherent to our study population. The prevalence of PPI-usage, chronic kidney disease, and other interacting factors in our control group was probably lower compared to the average hospital population. Moreover, the age of the control group was significantly lower compared to our patients; CgA is well-known to increase with age (42).

As for sensitivity, the high proportion of patients with stage IV disease in our population leads to a higher number of positive CgA results, as this is a measure of tumor burden (16). It is of clinical relevance that despite this high proportion of metastatic disease, CgA failed to accurately identify 44% of patients with neuroendocrine tumors. We compared healthy volunteers with patients known to have a NET. Therefore, strictly speaking, PPV, and NPV cannot be calculated. Nevertheless, we reported metrics like PPV and NPV because of the additional illustrative value. Furthermore, our population did not completely reflect age/gender-matched populations, but after stratification for age or gender, no differences in discriminating value or metrics were found.

NETest levels were not associated with tumor grade. This is in keeping with previous publications (26, 43). Although MKI67 is one of the markers measured by the NETest and both the NETest and tumor grade are independent predictors for disease progression (26, 44), the absence of an association is not surprising. MKI67 scores in blood correlate poor with tissue KI-67 because MKI67 is a dynamic tumor product capturing real-time biological variation whereas immunohistochemistry is a static pathological, “one-off” finding. Because the NETest consists of 6 different gene-clusters (hallmarks) covering a wider spectrum of neoplastic processes than only markers of proliferation (23), it is scientifically likely that there would be little to no association between the NETest and tumor grade.

Additionally, no association was found between tumor stage and NETest scores. This finding is in contrast with a previous publication (24). The small number of patients with loco-regional disease in this study might also have contributed to this difference. Nevertheless, a significant difference in NETest outcomes between the tumor stages in the functional imaging group was identified. However, it remains questionable if this indicates a true correlation with tumor load and behavior. The significant difference in the functional imaging subgroup could be the result of indication bias. Clinicians might prefer functional imaging over conventional imaging in case of clinically progressive disease to reassess tumor burden and behavior. When we take the function of the NETest into consideration (measure of biological activity) (23), it is possible that a higher proportion of patients with progressive disease is represented in the functional imaging group compared to the conventional imaging group, leading to a larger proportion of high NETest results. Other studies, using quantification of blood derived free-circulating nucleic acids as biomarker in malignancies, showed no correlation as well with tumor location, stage, or size (36). The lack of correlation with tumor burden might however be of limited relevance in predicting treatment effects in NET patients. Effective treatments for progressive disease are directed at decreasing tumor activity with a possible subsequent impact on tumor size. Therefore, a biomarker only reflecting tumor burden will have an insufficient predictive value. One might hypothesize that tumor activity is a more significant parameter of tumor burden than purely tumor mass. Therefore, a test that assesses tumor biology may be a useful tool for defining treatment strategy rather than the assessment of size alone. With no significant differences in NETest results between low- and high tumor burden, it will be very interesting to see in a prospective study if those with low tumor burden and high NETest-scores are indeed progressive.

The low specificity and the absence of a correlation with tumor characteristics raises questions on the principal source of transcripts. The principal sources theoretically could be any nucleated cell, including circulating tumor cells and leukocytes, but also platelets and circulating (extracellular) RNA. The selection procedure of the 51 marker genes, the fast decline in transcripts after surgery in bronchopulmonary NETs and the absence of a significant decline in transcripts after surgery in lung cancer patients seems to reflect a tumor-derived source of RNA (31, 45). However, the high proportion of positive results in healthy volunteers in the present study suggests that the involved molecular pathways reflected by the 51 marker genes might be activated in non-malignant processes as well.

In conclusion, this large independent validation study showed the NETest to be more sensitive, but less specific than Cga. The ability of the NETest to discriminate patients with GEP-NETs from controls was significantly better than CgA but given the low specificity, the NETest seems not suitable as a screening tool. The NETest was not associated with the origin of tumor, tumor grade and tumor load, leading to positive outcomes in low volume tumors as well. This creates a potential to combine the NETest with other independent markers of disease into a risk score or nomogram to provide not only a sensitive but also a specific diagnostic tool for better discrimination of those who are sick from healthy individuals. The superior sensitivity of the NETest over CgA in this study also supports the clinical potential of the NETest as a surveillance marker and as an indicator for residual disease after surgery. Validation of the predictive and prognostic value of the NETest in this Dutch cohort is underway to further assess its diagnostic advance in the management for neuroendocrine tumors.

## Ethics Statement

This study was carried out in accordance with the recommendations of Netherlands Cancer institute (NCI) local ethics committee with written informed consent from all subjects. All subjects gave written informed consent in accordance with the Declaration of Helsinki. The protocol was approved by the Netherlands Cancer institute (NCI) local ethics committee.

## Author Contributions

MvT performed the analysis and wrote the manuscript with input from all authors. MT, CK, LS, and WV collected all samples. MT and GV supervised the project. All authors discussed the results and contributed to the final manuscript.

### Conflict of Interest Statement

The authors declare that the research was conducted in the absence of any commercial or financial relationships that could be construed as a potential conflict of interest.
